# The surgical management of triple mandibular fractures: A challenging task

**DOI:** 10.4317/jced.62114

**Published:** 2024-10-01

**Authors:** Paolo Boffano, Francesca Neirotti, Panagiotis Stathopoulos, Muhammad Ruslin, Matteo Brucoli

**Affiliations:** 1 MD DDS. Division of Maxillofacial Surgery, University Hospital “Maggiore della Carità”, University of Eastern Piedmont, Novara, Italy; 2 MD. Division of Maxillofacial Surgery, University Hospital “Maggiore della Carità”, University of Eastern Piedmont, Novara, Italy; 3 MD. KAT Hospital, Athens, Greece; 4 Hasanuddin University, Makassar, Indonesia

## Abstract

**Background:**

The treatment of multiple mandibular fractures may often be challenging. The aim of this study was to evaluate the characteristics and outcomes of multiple mandibular fractures, with a focus on triple mandibular fractures.

**Material and Methods:**

Patients with multiple (triple) mandibular fractures were included. The following data were recorded for each patient: age; gender; cause of injury; sites of mandibular fractures; presence of complications. Facial width was esthetically evaluated through a clinical examination considering the inferior facial width, postoperative asymmetries, and facial esthetic harmony.

**Results:**

From January 1, 2010, to December 31, 2018, 25 patients (15 men, 10 women) underwent surgery for triple mandibular fractures and were included in the study. A predominance of symphysis/parasymphysis fracture associated with bilateral condylar/ramus fractures was observed, followed by symphysis/ parasymphysis fracture combined with mandibular angle fracture and condyle fracture. Most patients did not show any type of complications. An optimal esthetic outcome was obtained in 20 patients.

**Conclusions:**

A successful treatment of trifocal mandibular fractures may be achieved by different techniques, although it remains challenging. The re-establishment of the transversal bigonial dimension by a correct reconstruction of the mandibular arch should guide surgeons. The aim of the treatment should always be the successful rehabilitation of patients’ pretraumatic occlusion and function.

** Key words:**Mandibular fractures, multiple, treatment, management, mandible.

## Introduction

Mandibular fractures are among the most frequent fractures in maxillofacial trauma (1-5). Such injuries may often result in important functional damage and aesthetic loss with social repercussion (6-13). In particular, multiple (more than two sites) mandibular fractures involve more than two sites and are associated with high energy trauma: they require a precise treatment with a sTable fixation. The number of involved sites is crucial for the choice of the most appropriate treatment and for its outcomes, remembering that all patients with multiple mandibular fractures need surgical intervention (1,5,8,14-19).

The overall goal in the treatment of mandibular fractures is the re-establishment of the pre-injury occlusion as well as the achievement of an optimal functional and anatomic restoration. This objective may be remarkably difficult, however, in patients with multiple mandibular fractures (1-3,20-26). In fact, the post-traumatic augmented transverse dimension of the mandible is often encountered in patients with multiple mandibular fractures, thus modifying the inferior facial width. All these elements contribute to make the treatment of multiple mandibular fractures challenging.

In spite of the interesting and challenging issues that are associated with this peculiar trauma condition, in the literature, few articles have been published regarding multiple mandibular fractures. Therefore, the aim of this study was to evaluate the characteristics and outcomes of multiple mandibular fractures, with a focus on triple mandibular fractures.

## Material and Methods

The medical records of patients treated for mandibular fracture, between 2010 and 2018, were reviewed.

Panoramic radiographs, posteroanterior cephalograms, and head computed tomograms with 3-dimensional (3D) reconstruction were used to diagnose fractures. The treatment protocol included hospital admission followed by operative treatment under general anesthesia. After the application of maxillomandibular fixation screws or arch bars, the reduction of the fracture of the symphyseal region (or the body region, if a symphyseal fracture was absent) was carried out through an intraoral anterior degloving approach allowing the visualization of the inferior border, or through an extraoral laceration in the symphyseal region (if it was present). The symphyseal fracture was reduced thanks to a pressure applied to both gonial angles by the assistant surgeon and an adequate lingual fracture reduction was confirmed by visual and/or tactile examination. Then, the symphyseal fracture was fixed with miniplates that were bent to apply compression to the lingual cortex and eliminate the tendency for gap formation, as described by Ellis and Tharanon (2). If a body fracture was present (instead of a symphyseal fracture), the reduction was performed via the alignment of the mandibular fragments with the occlusion guide, and osteosynthesis was performed by two miniplates.

Then, distal (angle, ramus and subcondylar/condylar) fractures were treated by open reduction and internal fixation. Appropriate preoperative antibiotic therapy was given and was followed by 1 week of postoperative antibiotic therapy. The postoperative protocol for all patients included guidance elastics for 7 days, the use of elastics during the night for an additional 7 days, a soft diet for 30 days, functional exercises from day 15 onward, and the intensification of functional therapy from day 30 to at least 6 months after surgery.

The inclusion criteria were patients with multiple (triple) mandibular fractures. The exclusion criteria were the presence of previous mandibular fracture, edentulous patients, patients with bone-related diseases, incomplete records, or without follow-up. The following data were recorded for each patient: age; gender; cause of injury; sites of mandibular fractures; presence of complications (wound dehiscence, inferior alveolar nerve (IAN) impairment, infection, malocclusion).

Facial width was esthetically evaluated through a clinical examination considering the inferior facial width, postoperative asymmetries, and facial esthetic harmony, according to Gerbino *et al*. (1) by two surgeons (P.B; F.N.). Patients were rated as “excellent” (reestablished pre-traumatic appearance), “good” (presence of small defects and increased facial width), or “unsatisfactory” (major defects and clear evidence of increased facial width, which need a successive intervention).

The data were evaluated by means of descriptive statistics.

## Results

From January 1, 2010, to December 31, 2018, 25 patients (15 men, 10 women) underwent surgery for triple mandibular fractures and were included in the study.

**Figure 1:**
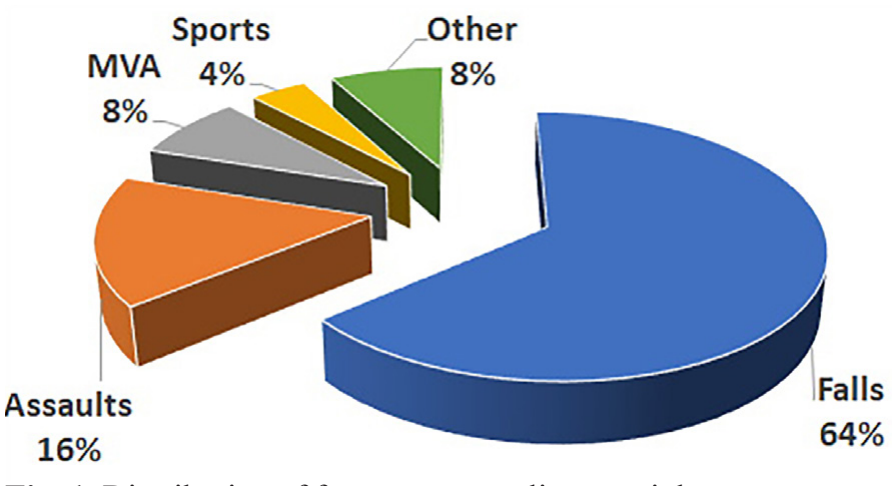
Distribution of fractures according to etiology.

The average age of the study population was 36.24 years (range 6 to 80 years). Most fractures resulted from falls (64%), followed by assaults (16%) (Fig. 1).

**Figure 2:**
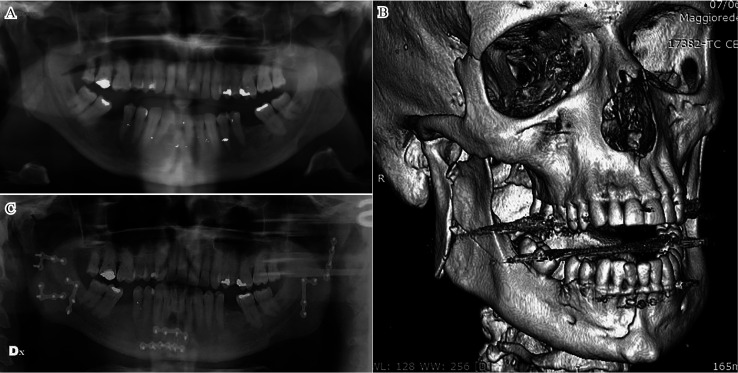
Preoperative panoramic radiograph (A), preoperative 3D CT scans (B), and postoperative panoramic radiograph (C) of a patient with triple mandibular fracture.

A predominance of symphysis/parasymphysis fracture associated with bilateral condylar/ramus fractures (Fig. 2) was observed, followed by symphysis/ parasymphysis fracture combined with mandibular angle fracture and condyle fracture.

 Table 1 describes the distribution of involved sites of fractures within the study population.

The time between trauma and treatment ranged from 0 to 5 days, with a mean of 2.1 days.

Most patients (22 out of 25) did not show any type of complications. The remaining 3 cases showed transitory inferior alveolar nerve (IAN) impairment (2 patients) and a wound dehiscence.

As for the esthetic evaluation of facial width according to Gerbino *et al*. (1), 20 patients were rated as excellent, 5 were rated as good, whereas no patient was rated as unsatisfactory.

## Discussion

The treatment of mandibular fractures may be complex, especially in cases with multiple mandibular fractures. Within our study population, both the male predominance and the role of falls confirm the results from the recent literature. The pathophysiology of mandibular fractures shows the role of the cause to the pattern and site of the fractures. Assaults usually produce angle or body fractures, whereas falls are typically responsible for symphyseal and condylar fractures, as it was observed in our study (1-4,8-14).

Some anatomical features, such as foramens, or the presence/absence of teeth decrease the mandible resistance and increase the susceptibility to fractures. Furthermore, bilateral fractures are more likely to occur due to the mandibular biomechanics. It is acknowledged that mandibular fractures occur in two ways: directly in the site of trauma, and indirectly in the contralateral region (1-3,6-10,13-19). The distribution of triple mandibular fractures within our study population confirms this theory.

As aforementioned, the treatment of multiple mandibular fractures is much more complex than that of isolated fractures. Our management protocol included the initial treatment of the most mesial fracture, followed by that of distal fractures. Of course, surgeons have to be guided by the principles of internal fixation for an appropriate decision-making process. In particular, the increase in the bigonial mandibular dimension by altering inferior facial width may be a challenging issue. In fact, the loss of continuity in the symphyseal region may determine mandibular retropositioning and splaying of the angles. The action of the suprahyoid, masseter, and temporal muscles overwhelm the antagonist pterygoid internal muscles. Furthermore, when condylar fractures are associated with symphyseal fractures, the splaying mechanism is worsened.

Therefore, the reestablishment of a correct transversal dimension while considering the bigonial diameter and mandibular base is crucial for a successful treatment in order to obtain an anatomic 3D reduction of the mandibular base arch (1,4,7,10,13,16,20,25).

Various methods for the rigid fixation of symphyseal fractures have been proposed in the literature (1,4,7,10,13,16,20,25) as treatments have evolved. In such cases, surgeons have to restore the correct transversal dimension while considering the bigonial diameter and mandibular base. Reduction is performed manually or with the use of elevators or bone hooks, as according to AO Surgery Reference, in addition to the application of high hand force at the gonial angles together with forward traction of the symphyseal region. The 3D perception of anatomic reduction is fundamental when an occlusal benchmark is unreliable or absent due to dental avulsions. As for the fixation system, AO Surgery Reference confirms the possibility to use various options of Open Reduction and Internal Fixation in not atrophic mandibles: lag screws, one load sharing plate, and two load sharing plates. At our center, we prefer to use two 2.0 miniplates although 2.3 plate remain a valid option. Of course, the contention at the dental level using arch bars or wires is fundamental.

The analysis of the results showed that it is possible to obtain adequate morphologic results in patients with multiple mandibular fractures. We chose to include triple mandibular fractures only, in order to grant the better uniformity to the study population (1-8,14-19).

In conclusion, successful treatment of trifocal mandibular fractures may be achieved by different techniques, although it remains challenging. The re-establishment of the transversal bigonial dimension by a correct reconstruction of the mandibular arch should guide surgeons. The aim of the treatment should always be the successful rehabilitation of patients’ pretraumatic occlusion and function.

## Figures and Tables

**Table 1:** Fracture sites in the study population.

Sites	Number of patients	Percentage
Para/symphysis–condyle bilateral	12	48%
Para/symphysis–angle–condyle	4	16%
Body–condyle bilateral	3	12%
Body–angle–condyle	2	8%
Symphysis–body–condyle	1	4%
Body–condyle–coronoid	1	4%
Para/symphysis– angle bilateral	1	4%
Para/symphysis– body bilateral	1	4%
TOTAL	25	100%

## Data Availability

The datasets used and/or analyzed during the current study are available from the corresponding author.
